# Comparative evaluation of root canal morphology in mandibular first premolars with deep radicular grooves using direct vision, dental operating microscope, 2D radiographic visualisation and micro-computed tomography

**DOI:** 10.1371/journal.pone.0329439

**Published:** 2025-07-31

**Authors:** Mohmed Isaqali Karobari, Hany Mohamed Aly Ahmed, Mohd Fadhli Bin Khamis, Norliza Ibrahim, Tahir Yusuf Noorani

**Affiliations:** 1 Department of Conservative Dentistry and Endodontics, Saveetha Dental College and Hospitals, Saveetha Institute of Medical and Technical Sciences, Saveetha University, Chennai, Tamil Nadu, India; 2 Department of Restorative Dentistry and Endodontics, Faculty of Dentistry, University of Puthisastra, Phnom Penh, Cambodia; 3 Department of Restorative Dentistry, Faculty of Dentistry, Universiti Malaya, Kuala Lumpur, Malaysia; 4 Oral Biology and Forensic Odontology Unit, School of Dental Sciences, Universiti Sains Malaysia, Health Campus, Kota Bharu, Kelantan, Malaysia; 5 Department of Oral and Maxillofacial Clinical Sciences, Faculty of Dentistry, Universiti Malaya, Kuala Lumpur, Malaysia; 6 Conservative Dentistry Unit, School of Dental Sciences, Universiti Sains Malaysia, Health Campus, Kota Bharu, Kelantan, Malaysia; Universidade Federal Fluminense, BRAZIL

## Abstract

**Objectives:**

The objective of this study was to evaluate the effectiveness of clinically applicable diagnostic methods such as Direct Vision (DV), Dental Operating Microscope (DOM), and two-dimensional (2D) radiographs in detecting canal orifices and root canal configurations in mandibular first premolars with deep radicular grooves. The study also aimed to compare these methods with micro-computed tomography (micro-CT) to better understand their diagnostic capabilities and to assess the applicability of the Ahmed et al. (2017) classification system across different imaging modalities.

**Methods:**

A total of 133 mandibular first premolars with deep radicular grooves were scanned using micro-CT. After scanning, two examiners, blinded to micro-CT results, accessed and examined the root canal orifices of the study samples under DV, and DOM. Further evaluation of the root canal configurations was undertaken using exploration and 2D radiographic images. A second round of relocation of canals was undertaken if the radiographic view showed the presence of a missed canal. The data were then compared with the original reconstructed micro-CT images. Descriptive statistical analysis, Kappa and chi-square tests were performed to compare between methods and examiners, and the *p*-value was set at 0.05.

**Results:**

Except for three teeth, all samples exhibited multiple canals. Micro-CT data revealed a wide range of root canal configurations, with the most common being ^1^MFP^1-2^ (19.54%), followed by ^1^MFP^1-2-3^ (15.78%), ^1^MFP^1-2-3-2^ (5.26%), and both ^1^MFP^1-2-4^ and ^1^MFP^1-2-1^ configurations, each at 4.51%. For both examiners, using the DOM resulted in detection of more canal orifices compared to DV (p < 0.001). A number of root canal configuration types including ^1^MFP^1-2^ and ^1^MFP^1-2-1^ was identified using exploration and radiographic imaging. Both examiners identified additional canals in the relocation phase. A significant difference was found when the radiographic method is compared with micro-CT results (*p* < 0.001), in which the latter method showed more complex root canal configuration types.

**Conclusions:**

Mandibular first premolars with deep proximal grooves exhibit considerable variability and complexity in their root canal systems. The use of a dental operating microscope (DOM) significantly improves the detection of canal orifices compared to direct vision. The most frequently observed root canal configuration was ^1^MFP^1-2^. A high percentage of additional canals may be missed due to atypical locations of bifurcations along the root. While micro-CT imaging is not applicable in clinical settings, it remains a valuable reference tool in studies for enhancing our understanding of root canal morphology and guiding the development of clinically feasible diagnostic strategies.

## Introduction

A comprehensive knowledge of the anatomy of roots and canals and their variations is essential for successful root canal treatment [1,2]. Mandibular first premolars are usually single-rooted with one or two canals [3]. However, literature shows that single-rooted mandibular first premolars may show a wide range of radicular grooves (RG) with a prevalence reaching more than 39% [4,5]. According to the Arizona State University Dental Anthropology Scoring System (ASUDAS), mandibular premolar teeth with radicular grooves have different morphological features, in which the deeper forms usually show complex root canal systems, including C-shaped canals and canal bifurcations at different levels of the root [4,6].

Along the years, several methods have been used to assess the internal and external tooth anatomy. These include clearing techniques, sectioning methods, 2D radiographic imaging, and 3D tomographic scans [7]. The high-resolution, non-invasive micro-computed tomography (micro-CT) has been used to provide detailed qualitative and quantitative analysis of the root canal anatomy [8]. Micro-CT has been a useful tool to characterize and analyse the root and canal anatomy of mandibular premolars with deep radicular grooves including the depth of the groove, root dentine thickness, root canal configurations, and accessory canals [4,5,9].

Access cavity preparation is defined as “the opening prepared in a tooth to gain entrance to the root canal system for cleaning, shaping, and obturating” [10]. One of the aims of access cavity preparation is to get direct straight-line access to the apical foramen, which helps to determine the root canal morphology and its variation. Sufficient access to the root system is required to carry out the biomechanical preparation of a root canal effectively [11,12]. An appropriate access cavity design, careful canal exploration and 2D radiographic imaging with proper angulations provide information about the possible locations of the root canal orifices and bifurcations. This information is essential to ensure the success and good prognosis of the treatment [13].

Although previous research has documented anatomical variations in mandibular first premolars using advanced imaging techniques [3,4,14], clinical diagnosis often relies on more accessible tools such as DV, DOM, and conventional 2D radiography. However, these clinical methods may fail to detect additional canal orifices or the full extent of internal morphological complexity. In contrast, micro-CT offers highly detailed, three-dimensional, non-destructive imaging and serves as the gold standard for *ex vivo* tooth morphology analysis. By comparing the findings of DV, DOM, and 2D radiography against micro-CT, this study aimed to assess the diagnostic performance and limitations of tools commonly used in clinical settings. The objective of this study was to evaluate the effectiveness of clinically applicable diagnostic methods such as DV, DOM, and 2D radiographs in detecting canal orifices and root canal configurations in mandibular first premolars with deep radicular grooves. The study also aimed to compare these methods with micro-CT to better understand their diagnostic capabilities and to assess the applicability of the Ahmed et al. [15] classification system across different imaging modalities.

## Materials and methods

### Ethical approval and study samples

Ethical approval was obtained from the Human Research Ethics Committee (JEPem) of Universiti Sains Malaysia (USM) with ethical approval code number USM/JEPeM/21070530. The study was carried out following the Declaration of Helsinki. The study employed a multifaceted design incorporating laboratory-based micro-CT analyses conducted at the Faculty of Dentistry, Universiti Malaya, and clinical evaluations at the School of Dental Sciences, USM using the PRILE guidelines.

A total of 133 human mandibular first permanent premolars with deep radicular grooves were collected over the period from December 2021 to October 2022 and the soft tissues and dental calculus were removed using an ultrasonic scaler (Dentsply Sinora, Bensheim, Germany). The teeth were stored in 5.25% sodium hypochlorite (NaOCl, Lenntech, Delfgauw, The Netherlands) for 30 mins and reserved in 10% neutral buffered formalin to dissolve any tightly adherent contaminants. Subsequently, the samples were stored in distilled water until the time of testing. To reduce deterioration, the storage medium was replaced every alternate day.

### Inclusion and exclusion criteria

This study included mandibular first premolar teeth extracted for various reasons not related to the study. Mandibular first premolars with complete roots and exhibiting deep radicular grooves [Grades 2, 3 and 4 according to the Arizona State University Dental Anthropology Scoring System (ASUDAS) scoring (Turner, [16)] were included. The radicular grooves were categorized as follows:

Grade 0: A radicular groove is absent; if present, it is shallow with rounded indentation.
Grade 1: A radicular groove is present and has a shallow V-shaped cross-section.
Grade 2: A radicular groove is present and has a moderately deep V-shaped cross-section.
Grade 3: A radicular groove is present and has a markedly deep V-shaped cross-section, such that the radicular groove extends to at least 1/3^rd^ of the total root length.
Grade 4: A radicular groove is deeply invaginated on mesial and distal root surfaces.
Grade 5: Two independent roots are present, so their length is at least 1/4^th^ to 1/3^rd^ of the total root length.

The exclusion criteria were study samples with incomplete root development, root fractures, significant root caries, internal resorption, and those with previous root canal treatment.

### Sample size calculation

The sample size was calculated using a sample size calculator (https://select-statistics.co.uk/calculators/sample-size-calculator-population-proportion/) with the following values: the margin of error was set at 5%, 90% for the confidence interval, 87% for the expected sample proportion, and the total population of Malaysia is approximately 32.8 million [17]. The minimum sample size was calculated as 123 specimens to achieve adequate statistical power. However, to account for possible exclusions due to specimen damage, imaging artifacts, or data loss during processing, a total of 133 mandibular first premolars were included in the study.

### Phase 1: Micro-CT scanning

The included samples were thoroughly examined for the inclusion and exclusion criteria using an ultra-view magnifying lens 4D (Toffeln, Bristol, United Kingdom). The samples were then divided depending on the presence of radicular grooves. The presence of grooves and further scoring (grade) was done based on the Arizona State University Dental Anthropology Scoring System (ASUDAS) scoring [16]. The teeth with deep grooves (i.e., grades 2, 3 and 4) and intact root tips were included in the current research [5].

The collected samples of mandibular first premolar teeth with deep radicular grooves were scanned using a high-resolution micro-CT system (X-ray, Xradia 520 Versa, Zeiss, Germany) at 65 kV, 692 mA, with a rotation step of 0.2°, 360° around the vertical axis, and 20 μm voxel size. The 3D reconstruction of the samples was performed after the second phase mentioned below.

### Phase 2: Characterization of the root canal morphology

#### a) Direct vision.

The access opening and assessment of the number of the root canal orifices were conducted by two examiners, who were blinded to the data obtained from micro-CT analysis. A standardised occlusal access cavity was prepared to approach the occlusal surface using tapered diamond bur with 0.9 mm diameter (Dentsply Maillefer, USA) with high-speed handpiece following the standardisation of the pulp chamber anatomy [18]. A long non-end cutting bur (#851, Dental Burs Australia Pty. Ltd., Australia) was used with a low-speed handpiece to smoothen the walls of the cavity and ensure the roof of the pulp chamber was removed entirely and free from dentine ledges [19,20]. Following the access cavity preparation, 5.25% NaOCl irrigation was performed using a 5 mL syringe and a NaviTip 30-G (Ultradent, South Jordan, UT, USA) needle to improve visualization of the pulp chamber floor. Barbed broaches (sizes 15-40, Dentsply, Switzerland) were used to extirpate the pulp tissue. Root canal patency was evaluated using Stainless steel (SS) K-files sizes 06 and 08 (Dentsply/Maillefer, Ballaigues, Switzerland). The inspection was performed without magnification.

#### b) Dental operating microscope.

The DOM (S100/ OPMI Pico from ZEISS, Germany), equipped with a foldable tube f170 with PROMAG function, an integrated camera control unit (full HD 1080p), and a focusing objective lens f=250, was used at magnifications of 6x, 10x, and 14x. The orifices were examined and counted by two examiners and then compared to the DV method.

#### c) 2D Radiographic assessment.

Initially, a pilot study was conducted using 20 samples to check the best angulation for evaluating root and canal morphology in mandibular first premolars. For the 2D radiographic assessment, SS K-files (Dentsply/Maillefer, Ballaigues, Switzerland) in sizes 10, 15, 20, and 25 were used. These files were positioned within the canals during the radiographic imaging process to enhance the visualization of the root canal pathways across the different angulations of the X-ray cone. A model was prepared with different angulations starting from zero, 20 and 40 degrees of angulation of the cone to the long axis of the tooth. The model system consisted of a marked place for a saw dust mould holding the extracted tooth, a place to hold the sensor, and the marking for different angulations, zero, 20, and 40 degrees, using a geometric protector. A mark was made at the center of the cone, which was in turn made to coincide with the different angulations marked on the model as mentioned earlier and a radiograph was taken. Three radiographs were taken for each sample to assess and compare the root canal morphology.

The radiographs were taken using Ezsensor Vatech 1.5 (Gyeonggi-do, Korea) and tube generator housing assembly FOCUS tube head serial number 42657 T (Tussula, Finland) having an exposure of 70 kV, 8 mA with total filtration of 2.0 mm Al. The radiographic images were analysed using Ezdent-I version 3.1.11.1 (Ewoosoft Co, Ltd) in a BARCO medical radiology system (Kennedypark 35,8500 Kortrijk, Belgium), model number MDRC 2224 with an LED 24“screen, resolution 1920 x 1200 pixels with a pixel pitch of 0.270 x 0.270 mm; the system was inbuilt with an Intel C236 chipset and the screen with a front sensor, in a dark room.

**Fig 1:**
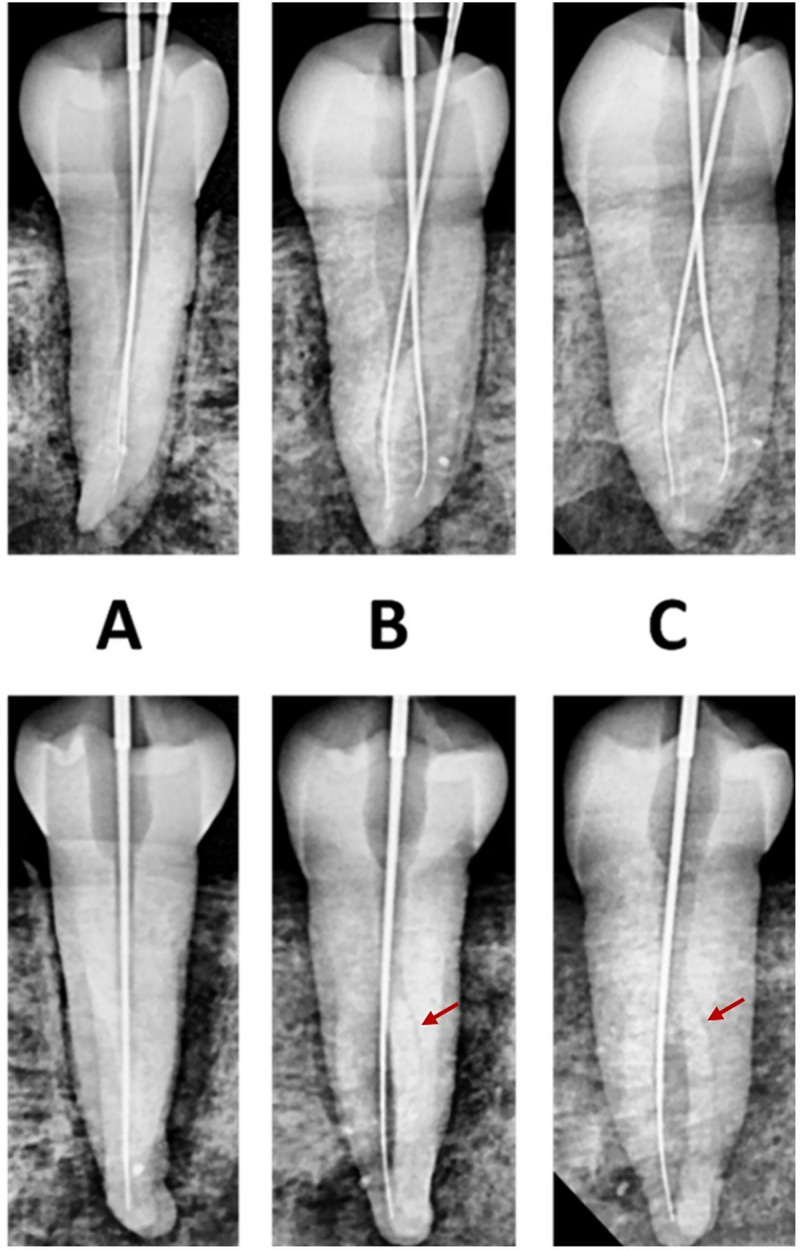
Radiographic images taken at different angulations in the pilot study showing detection (above) and missing of canals (below): A) at zero degrees, B) at 20 degrees, and C) at 40 degrees. Red arrow showing the missed canals.

The images taken from all three different angulations were assessed, and the images taken at 40 degrees to the long axis of the tooth showed the best root canal morphology of mandibular first premolars (Fig 1). Consequently, all the samples were radiographed at 40-degree angulation and examined using the above software. Two examiners classified and recorded root canal morphology using Ahmed et al. [15] coding system. The determined root canal morphology using radiographs was compared with micro-CT.

#### d) Reconstruction of the micro-CT scanned teeth.

After data collection of the 2D radiographic imaging, the DICOM files of the previously micro-CT scanned teeth (before intervention) were reconstructed into a 3D dataset using the Mimics software version 21.0 (Materialise, Leuven, Belgium) (Figs 7–9). Ahmed et al. [15] coding system was used for the classification of the root canals. A calibration was undertaken between the two observers on 12 samples. Disagreements were discussed, and a consensus was reached after discussion.

**Fig 2:**
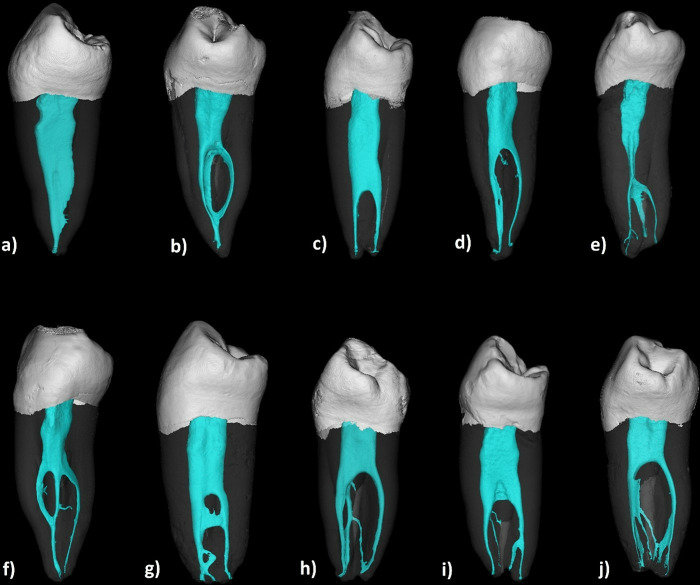
Reconstructed 3D images from micro-CT scanned mandibular first premolars showing different codes. a) ^1^MFP^1^, b) ^1^MFP^1-2-1^, c) ^1^MFP^1-2^, d) ^1^MFP^1-2-3-2^, e) ^1^MFP^1-3^, f) ^1^MFP^1-3-2^, g) ^1^MFP^1-2-1-3-2-3^, h) ^1^MFP^1-2-3-4-2^, i) ^1^MFP^1-2-4^, j) ^1^MFP^1-2-3-5^.

**Fig 3:**
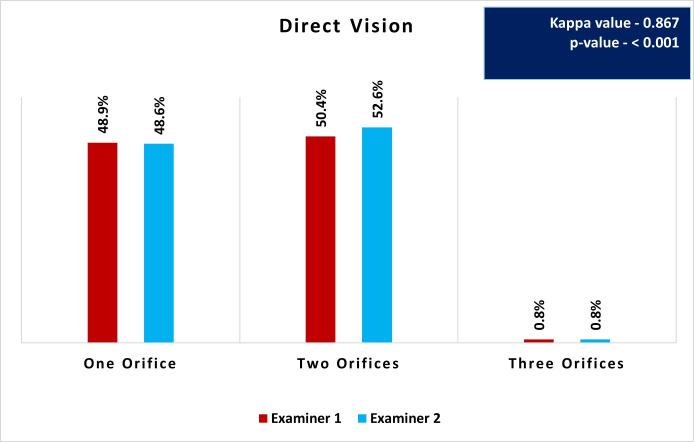
Distribution and comparison of root canal orifices using direct vision by Examiners 1 and 2.

**Fig 4:**
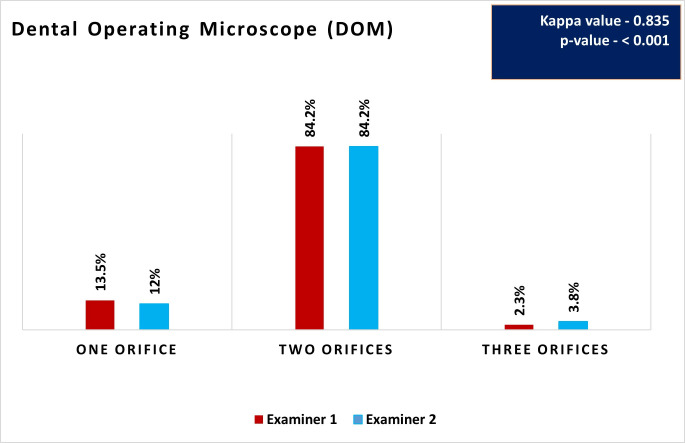
Distribution and comparison of root canal orifices using DOM by Examiners 1 and 2.

**Fig 5:**
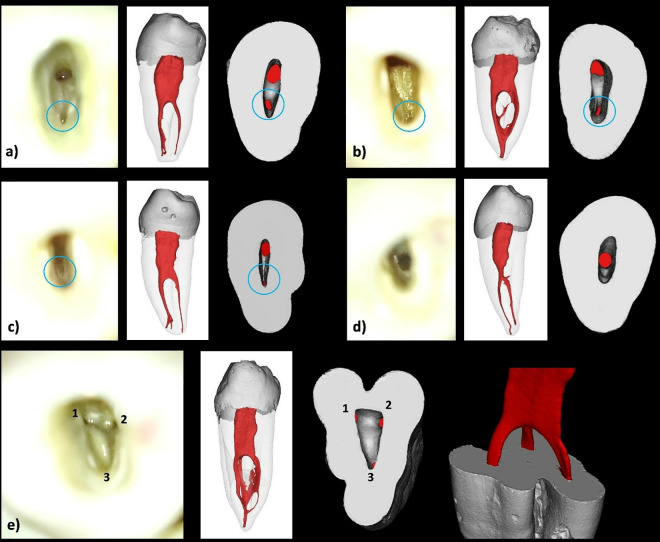
Samples showing the detection of root canal orifices under the DOM. a-c) Successful detection of the buccal and lingual canal orifices. **d)** Detection of only one orifice in a sample with a bifurcation in the middle third of the root. **e)** Detection of 3 root canal orifices.

**Fig 6:**
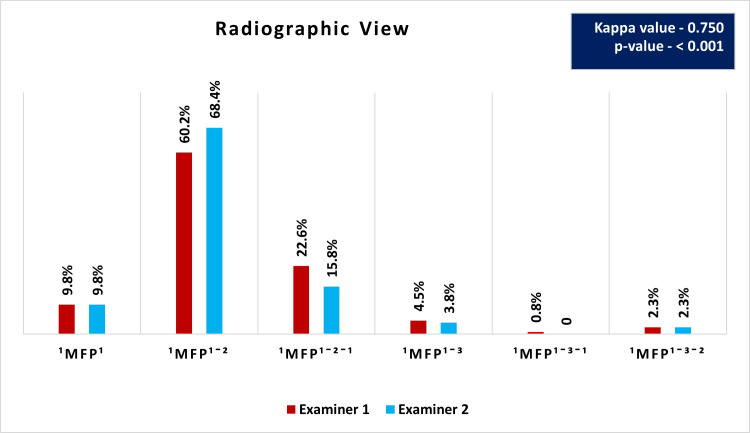
Distribution and comparison of root canal morphology using 2D radiography by Examiners 1 and 2.

**Fig 7:**
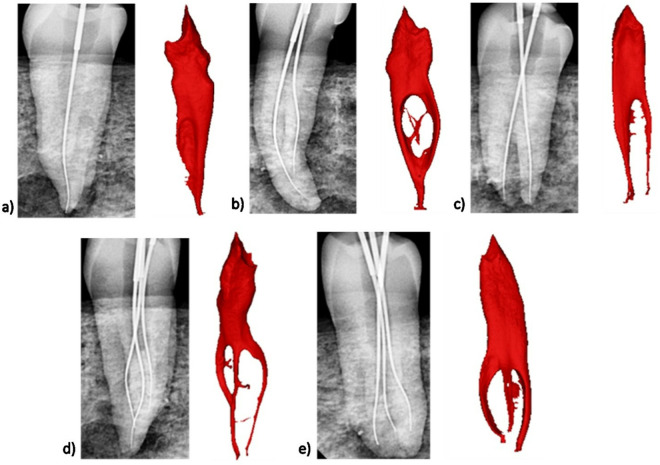
Detection of the root canal configuration using radiographic imaging in comparison with the micro-CT. a) ^1^MFP^1^, b) ^1^MFP^1-2-1^, c) ^1^MFP^1-2^, d) ^1^MFP^1-3-2^, e) ^1^MFP^1-3^.

**Fig 8:**
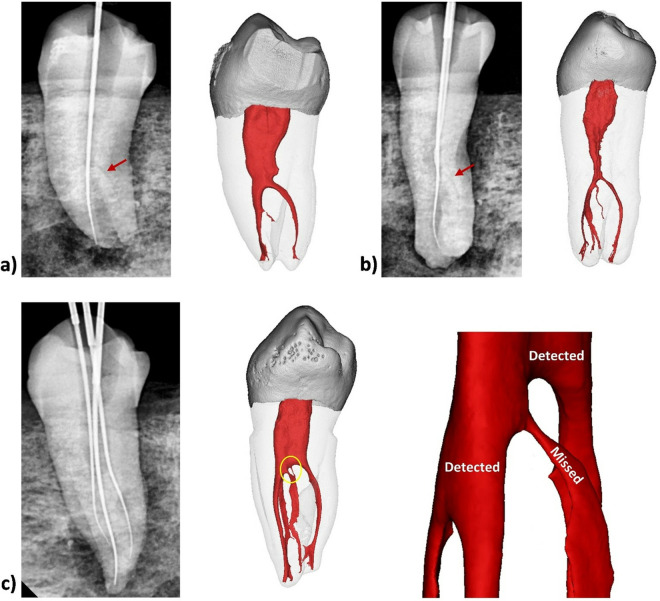
Samples showing the root and canal morphology classification in mandibular first premolars. Radiographic view (Arrow showing missed canal) and Reconstructed 3D images from micro-CT scanned mandibular first premolars showing missed and detected canals.

**Fig 9:**
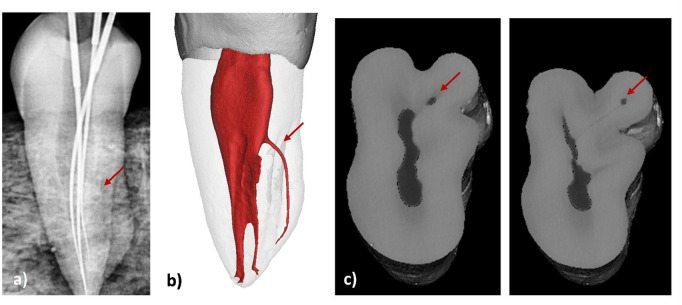
Samples showing the root and canal morphology classification in mandibular first premolars. A) Radiographic view (Arrow showing missed canal), B) and C) Reconstructed 3D images from micro-CT scanned mandibular first premolar (Arrow showing extra canal).

### Statistical analysis

Descriptive statistical analysis was done using the software SPSS version 26 [IBM SPSS statistics], and the frequency, mean, and standard deviation were calculated. The chi-square test was done for association and significance value was set at 0.05. The Kappa and Chi-square tests were done to identify the significant difference between the two examiners. Kappa statistics was interpreted as following: less than 0.2 represents slight agreement; 0.2–0.4 represents fair agreement; 0.41–0.6 represents moderate agreement; 0.61–0.8 represents substantial agreement; greater than 0.8 represents almost perfect agreement [21].

## Results

### Root canal configuration using micro-CT

Table 1 provides the distribution of different configurations observed and their frequencies and percentages. Except for three teeth, all samples encased more than one canal. The most common configuration, ^1^MFP^1-2^ was identified in 26 samples, making up 19.54% of the total sample. This code indicates a single-rooted mandibular first premolar with one canal orifice that bifurcates into two separate canals. ^1^MFP^1-2-3^ was observed in 21 samples (15.78%) which represents one orifice dividing into two canals and then trifurcating into three canals. The code ^1^MFP^1-2-3-2^ was found in seven samples (5.26%) denoting sequential a canal configuration of 1-2-3-2. The ^1^MFP^1-2-4^ and ^1^MFP^1-2-1^ configuration was found in six samples (4.51%) (Fig 2).

**Table 1:** Distribution and percentage of root canal morphology variants in mandibular first premolars according to Ahmed *et al.* (2017) classification (MFP -Mandibular First Premolars).

S/no	Configuration	Frequency (n)	Percentage (%)	S/no	Configuration	Frequency (n)	Percentage (%)
1	^1^MFP^1-2^	26	19.54	20	^1^MFP^1-2-1-2-1^	01	0.75
2	^1^MFP^1-2-3^	21	15.78	21	^1^MFP^1-2-5-4^	01	0.75
3	^1^MFP^1-2-3-2^	07	5.26	22	^1^MFP^1-3-2-4^	01	0.75
4	^1^MFP^1-2-4^	06	4.51	23	^1^MFP^1-3-5^	01	0.75
5	^1^MFP^1-2-1^	06	4.51	24	^1^MFP^1-4-5^	01	0.75
6	^1^MFP^1-3^	05	3.75	25	^1^MFP^1-4-3^	01	0.75
7	^1^MFP^1-2-3-2-4^	05	3.75	26	^1^MFP^1-4-3-5^	01	0.75
8	^1^MFP^1-2-1-2^	05	3.75	27	^1^MFP^1-4^	01	0.75
9	^1^MFP^1-2-1-2--1--2^	04	3.00	28	^1^MFP^1-2-1-3-2-3^	01	0.75
10	^1^MFP^1-2-3-5^	04	3.00	29	^1^MFP^1-2-3-1^	01	0.75
11	^1^MFP^1-2-5^	04	3.00	30	^1^MFP^1-2-3-2-1^	01	0.75
12	^1^MFP^1^	03	2.25	31	^1^MFP^1-4-5-4^	01	0.75
13	^1^MFP^1-2-3-2-3^	03	2.25	32	^1^MFP^1-2-3-1-2-1^	01	0.75
14	^1^MFP^1-2-4-6^	03	2.25	33	^1^MFP^1-2-5-4^	01	0.75
15	^1^MFP^1-2-3-4-3^	03	2.25	34	^1^MFP^1-3-4-3-6^	01	0.75
16	^1^MFP^1-2-1-3^	03	2.25	35	^1^MFP^1-2-1-2-1-3^	01	0.75
17	^1^MFP^1-3-1^	02	1.50	36	^1^MFP^1-2-3-1-2-1^	01	0.75
18	^1^MFP^1-3-2^	02	1.50	37	^1^MFP^1-3-1-2-1^	01	0.75
19	^1^MFP^1-2-3-4-2^	02	1.50	38	^1^MFP^1-2-3-4^	01	0.75
	**Total**	**114**	**85.71**			**19**	**14.29**

### Root canal orifices detection using direct vision

Fig 3 illustrates the distribution and comparison of root canal orifices detected by Examiner 1 and 2 using DV. The results showed that both examiners identified one orifice in approximately 48% of the cases (Examiner 1: 48.9%, Examiner 2: 48.6%). Two orifices were detected slightly more frequently, with Examiner 1 identifying them in 50.4% of the cases and Examiner 2 in 52.6%. Three orifices were the least detected configuration, with both examiners identifying them in only 0.8% of cases. The Kappa value of 0.867 indicates an almost perfect agreement between the two examiners.

### Root canal orifices detection using DOM

Fig 4 shows the distribution and comparison of root canal orifices identified by Examiner 1 and 2 using the DOM. Both examiners predominantly identified two orifices, with each detecting them in 84.2% of the cases (Fig 5). The detection of one orifice was less frequent, with Examiner 1 identifying it in 13.5% of the cases and Examiner 2 in 12%. The least detected configuration was three orifices, observed by Examiner 1 in 2.3% of cases and by Examiner 2 in 3.8% (Fig 5). The Kappa value of 0.835 indicates an almost perfect agreement between the two examiners.

### Comparison of detection methods (Direct vision vs DOM)

Table 2 shows the comparison of root canal orifices identified using direct vision and DOM by Examiners 1 and 2. The results shows a significant difference in the detection of root canal orifices between the two methods for both examiners (*p* < 0.001). Under DV, both examiners predominantly identified two orifices, but the use of the DOM significantly increased the identification of two and three orifices.

**Table 2:** Comparison of root canal orifices in mandibular first premolars recorded by examiners 1 and 2 (Direct vision vs DOM-Dental Operating Microscope).

Direct Visualization	Dental Operating Microscope				Chi-square value (d)	p-value
	One orifice	Two orifices	Three orifices	Total		
**Examiner 1**						
One orifice	18	47	0	65	66.051 (4)	**<0.001**
Two orifices	0	65	2	67		
Three orifices	0	0	1	1		
**Total**	**18**	**112**	**3**	**133**		
**Examiner 2**						
One orifice	16	44	2	62	46.682 (4)	**<0.001**
Two orifices	0	68	2	70		
Three orifices	0	0	1	1		
**Total**	**16**	**112**	**5**	**133**		

### Root canal configuration using 2D radiography

Fig 6 illustrates the distribution and comparison of root canal morphology identified through 2D radiography by Examiners 1 and 2. The most frequently observed configuration was ^1^MFP^1-2^, detected by Examiner 1 in 60.2% of cases and by Examiner 2 in 68.4% of cases. The next most common configuration, ^1^MFP^1-2-1^, was identified by Examiner 1 in 22.6% of cases and by Examiner 2 in 15.8% of cases. Both examiners observed the ^1^MFP^1^ configuration in 9.8% of cases each. Less frequent configurations like ^1^MFP^1-3^, ^1^MFP^1-3-1^, and ^1^MFP^1-3-2^ were identified in 4.5%, 0.8%, and 2.3% of cases by Examiner 1, respectively, and in 3.8%, 0%, and 2.3% of cases by Examiner 2, respectively. The Kappa value of 0.750 indicates a substantial agreement between the two examiners. Fig 7 shows root canal configurations identified in radiographic images compared to micro-CT. Figs 8 and 9 show samples where canals were missed during detection.

### Relocation of root canal configurations after radiographic imaging

Relocation refers to instances where the root canal configuration initially identified through 2D radiographs was revised after comparison with the corresponding micro-CT data. Examiner 1 identified such changes in 14 out of 47 teeth (29.8%), while Examiner 2 noted relocation in 21 out of 40 teeth (52.5%). This indicates that Examiner 2 identified a greater number of discrepancies between radiographic and micro-CT findings, reflecting a higher sensitivity to anatomical variations not captured on radiographs alone.

### Comparison of root canal morphology evaluation between radiograph and micro-CT

Tables 3 and 4 present the comparative analysis of root canal configurations identified by 2D radiographic imaging and micro-CT for both examiners. The direction of these analyses indicates a consistent trend: 2D radiographs significantly underreported complex root canal morphologies compared to micro-CT (χ², p < 0.001). Specifically, radiographs more frequently identified simpler configurations (e.g., single canals or unbranched types), whereas micro-CT revealed a higher prevalence of bifurcations and C-shaped canal systems. These discrepancies underscore the limited diagnostic sensitivity of conventional radiographic imaging when evaluating teeth with deep radicular grooves. Additionally, inter-examiner analysis revealed instances of disagreement in classification, particularly with radiographic interpretation. Consensus was reached after joint review and discussion.

**Table 3:** Distribution and percentage of radiograph view with micro-CT comparison for examiner 1 (MFP -Mandibular Fist Premolars) (numbers in bracket = percentage).

Micro-CT	Radiograph							χ^2^-value	df	p-value
	^1^MFP^1^	^1^MFP^1-2^	^1^MFP^1-2-1^	^1^MFP^1-3^	^1^MFP^1-3-1^	^1^MFP^1-3-2^	Total			
^1^MFP^1^	2	1	0	0	0	0	**3 (2.3)**	149.295	65	< 0.001*
^1^MFP^1-2^	3	16	7	0	0	0	**26 (19.5)**			
^1^MFP^1-2-1^	0	2	5	0	0	0	**7 (5.3)**			
^1^MFP^1-3^	1	2	1	0	0	0	**4 (3)**			
^1^MFP^1-3-1^	0	0	0	0	0	2	**2 (1.5)**			
^1^MFP^1-3-2^	0	0	2	0	0	0	**2 (1.5)**			
^1^MFP^1-3-5^	0	1	0	0	0	0	**1 (0.8)**			
^1^MFP^1-2-3^	1	16	1	1	0	0	**19 (14.3)**			
^1^MFP^1-2-5^	1	3	0	0	0	0	**4 (3)**			
^1^MFP^1-4-5^	0	1	1	0	0	0	**2 (1.5)**			
^1^MFP^1-2-4^	0	5	2	2	0	0	**9 (6.7)**			
^1^MFP^1-4-3^	0	0	1	0	0	0	**1 (0.8)**			
^1^MFP^1-4^	1	0	0	0	0	0	**1 (0.8)**			
^1^MFP^1*^	4	33	10	3	1	1	**52 (39)**			
**Total**	**13 (9.8)**	**80 (60.1)**	**30 (22.5)**	**6 (4.5)**	**1 (0.8)**	**3 (2.3)**	**133 (100)**			

*Significant p-value <0.05; ^1^MFP^1^* -canal variation having more than three-digit code

**Table 4:** Distribution and percentage of radiograph view with micro-CT comparison for examiner 2 (MFP -Mandibular First Premolars) (numbers in bracket = percentage %).

Micro CT	Radiograph						χ^2^-value	df	p-value
	^1^MFP^1^	^1^MFP^1-2^	^1^MFP^1-2-1^	^1^MFP^1-3^	^1^MFP^1-3-2^	Total			
^1^MFP^1^	2	1	0	0	0	**3 (2.3)**	146.075	52	< 0.001*
^1^MFP^1-2^	3	18	4	1	0	**26 (19.5)**			
^1^MFP^1-2-1^	0	2	5	0	0	**7 (5.3)**			
^1^MFP^1-3^	1	2	1	0	0	**4 (3)**			
^1^MFP^1-3-1^	0	0	0	0	2	**2 (1.5)**			
^1^MFP^1-3-2^	0	1	1	0	0	**2 (1.5)**			
^1^MFP^1-3-5^	0	1	0	0	0	**1 (0.8)**			
^1^MFP^1-2-3^	1	17	0	1	0	**19 (14.3)**			
^1^MFP^1-2-5^	1	3	0	0	0	**4 (3)**			
^1^MFP^1-4-5^	0	2	0	0	0	**2 (1.5)**			
^1^MFP^1-2-4^	0	8	0	1	0	**9 (6.7)**			
^1^MFP^1-4-3^	0	0	1	0	0	**1 (0.8)**			
^1^MFP^1-4^	1	0	0	0	0	**1 (0.8)**			
^1^MFP^1*^	4	36	9	2	1	**52 (39)**			
**Total**	**13 (9.8)**	**91 (68.4)**	**21 (15.8)**	**5 (3.7)**	**3 (2.3)**	**133 (100)**			

*Significant p-value <0.05; ^1^MFP^1^* -canal variation having more than three-digit code

## Discussion

Studying root and canal morphology is essential for successful endodontic treatment planning and execution [22]. Micro-CT provides a non-invasive and reliable method for quantitatively and qualitatively evaluating the 3-dimensional structure of the root canal system [14]. Recent advancements in imaging technology, particularly micro-CT, have opened up horizons for precisely characterising dental root and canal morphology [23]. Although both micro-CT and CBCT are three-dimensional imaging modalities, they serve different purposes. Micro-CT is a non-clinical, high-resolution diagnostic research tool used here as a gold standard for morphological assessment, whereas CBCT, though clinically available, offers lower resolution and was not evaluated in this study. Therefore, direct clinical recommendations involving CBCT cannot be derived from our micro-CT findings. In this study, micro-CT imaging and a recently introduced classification system developed by Ahmed *et al.* [15] were used to comprehensively characterise the root and canal morphology of mandibular first premolar teeth with deep radicular grooves. A recent systematic review has shown that this system is more accurate compared to other systems, especially when high resolution devices are used for analysis (such as micro-CT) [24]. In this study, this coding system was able to classify all samples with both 2D radiographic and micro-CT methods.

The *ex vivo* evaluation of mandibular first premolars is a critical bridge between laboratory findings and real-world dental practice. This study aimed to assess the root and canal morphology of mandibular first premolars under DV, DOM, and 2D radiographic images, comparing the latter with micro-CT data, which allowed translating research to practical clinical scenarios. This validation process enhances the clinical relevance of the findings and provides an opportunity to identify potential areas for improvement in detection of canals in teeth with complex canal anatomy.

Results from mirco-CT showed that ^1^MFP^1-2^ was the most prevalent configuration in the current study (19.54%), which aligns with previous research [4,5,25]. The ^1^MFP^1-2-3^ configuration was observed in 19 samples, accounting for 14.28%. This prevalence highlights the importance of thorough diagnostic imaging and clinical examination, as the additional canal may not always be apparent. Similar findings were observed in a study conducted among the Chinese population [26] for this configuration. More complex configurations, such as ^1^MFP^1-2-3-2^ and ^1^MFP^1-2-4^, were identified in 8 samples, representing 6.01% of the studied sample. Other configurations, such as ^1^MFP^1-2-1^, was observed in 5.26% of the samples, consistent with previous studies [5,27,28]. These variations in root canal morphology in mandibular first premolars could be multifactorial and can result from a combination of genetic, developmental, environmental, and evolutionary factors [29,30]. These findings highlight the limitations of conventional radiographic methods in detecting complex canal anatomy. While CBCT offers improved 3D imaging in clinical settings, it was not assessed in this study.

Results showed statistically significant differences for both examiners in their assessments under DV and DOM; this emphasizes the importance of using magnification for the detection of canals in this anatomical variation of mandibular premolars. These findings are consistent with previous studies [31–33], which have reported the superiority of DOM in improving the visualization of complex root canal anatomy. The magnification devices such as DOM significantly increase the detection of additional root canal orifices that are often missed under DV [34]. The increased detection of two and three orifices under the DOM can be attributed to the enhanced illumination and magnification, allowing for a more detailed examination of the pulp chamber floor [35]. This is particularly important in cases where the anatomy is complex or when the orifices are small and more difficult to detect without magnification. However, it has to be highlighted that not all root canal orifices/bifurcations can easily be detected using the DOM, even with the trained operator. Some of them can be too small, at different locations or bifurcating laterally from the main canal, which are the main reasons for missing canals in this study as shown in Fig 5d. In clinical scenarios, careful tactile exploration using small stainless-steel K-files, possibly supported by available 3D imaging modalities when clinically indicated, may improve the detection of additional canals.

The study findings revealed a significant agreement between the two examiners in detecting root canal orifices in mandibular first premolars using DV and DOM. Such agreement is essential in endodontic practice as it ensures multiple clinicians can reliably interpret clinical findings [36]. The high degree of agreement among examiners might be related to other factors, such as standardised examination criteria and protocol.

The canal detection and identification in 2D radiographic was performed in this study to simulate clinical settings. A significant difference was found between the radiographic view and micro-CT imaging which emphasizes the inherent limitations of 2D radiography in accurately detecting complex root canal anatomy compared to micro-CT. The findings of this study align with other research that demonstrated the limitations of 2D radiographs in accurately identifying root canal configurations [37–39]. While micro-CT provides high accuracy in identifying root canal configurations, it cannot be used clinically because of the high radiation dose and long exposure time [40,41]. Therefore, the operator should interpret carefully the 2D radiographic images exposed at different horizontal angulations to identify possible locations of canal bifurcations. Both examiners were able to locate additional canals after examining the radiographic images.

The results of this study should be interpreted with caution. Despite that micro-CT results showed different locations of small canal orifices that were not identified during exploration (Figs 8c and 9), the majority of samples showed different canal configurations from radiographic images because of the presence of complex intercanal communications/canal isthmus or apical canal bifurcations which cannot be detected clinically (Fig 2). Indeed, such parts of the canals cannot be instrumented and usually left for chemical disinfection using activated root canal irrigation protocols [42]. This is usually followed by warm compaction techniques to fill the complex pulp spaces between the main canals.

This study has limitations. The demographic data such as age, gender and ethnic groups of the subjects were not recorded. Future research is needed to explore the optimal instrumentation and root filling techniques for such complex canal anatomy in mandibular premolars with radicular grooves. The application of such detection methods (2D radiograph versus micro-CT) can be applied in other teeth such as maxillary and mandibular molars.

## Conclusions

Mandibular first premolars with deep proximal grooves exhibit considerable variability and complexity in their root canal systems. The use of a DOM significantly improves the detection of canal orifices compared to direct vision. The most frequently observed root canal configuration was 1MFP 1,2. A high percentage of additional canals may be missed due to atypical locations of bifurcations along the root. While micro-CT imaging is not applicable in clinical settings, it remains a valuable reference tool in ex vivo studies for enhancing our understanding of root canal morphology and guiding the development of clinically feasible diagnostic strategies.

## Supporting information

S1 File Data set. (XLSX)

S2 File PRILE-checklist. (DOCX)

S3 File PRILE-flowchart. (DOCX)
