# Correction: Comparative evaluation of root canal morphology in mandibular first premolars with deep radicular grooves using direct vision, dental operating microscope, 2D radiographic visualisation and micro-computed tomography

**DOI:** 10.1371/journal.pone.0349881

**Published:** 2026-05-26

**Authors:** Mohmed Isaqali Karobari, Hany Mohamed Aly Ahmed, Mohd Fadhli Bin Khamis, Norliza Ibrahim, Tahir Yusuf Noorani

The first sentence of the third paragraph in the Discussion should have cited references 5, 14 and 25 instead of 4, 5 and 25.

The correct sentence should read: Results from mirco-CT showed that ^1^MFP^1-2^ was the most prevalent configuration in the current study (19.54%), which aligns with previous research [5, 14, 25‌].

There is an error in reference 15, 16, 17. The correct references are.

15. Ahmed HMA, Versiani MA, De-Deus G, Dummer PMH. A new system for classifying root and root canal morphology. International Endodontic Journal 2017. 50(8): p.761–770
16. Turner CI. Scoring produces for key morphological traits of the permanent dentition: The Arizona State University dental anthropology system. Adv Dent Anthropol. 1991:13–31.
17. Malaysia, D.O.S., Department of Statistics Malaysia, Press Release Current Population Estimates, Malaysia. 2020. Available from: https://www.dosm.gov.my/v1/index.php?r=column/pdfPrev&id=OVByWjg5YkQ3MWFZRTN5bDJiaEVhZz09
